# Bohemian Veterinary Institution at Prague

**Published:** 1896-09

**Authors:** 


BOHEMIAN VETERINARY INSTITUTION AT PRAGUE. At the session of the Bohemian Landtag, on January 17, 1896, the motion of Representative Dwoîak, in regard to the establishment of a veterinary school at Prague, came up for the first reading. The motion was: The Provincial Committee is empowered to open negotiations with the Imperial Government as soon as possible in order that a veterinary school for the Kingdom of Bohemia may be founded at Prague, in order to meet the long-felt need of veterinarians qualified in all branches of their profession. Representative Dwoîak, in putting the motion, spoke of the reform of veterinary studies, the necessity of prolonging the course to four years, and of raising the institution to the dignity of a high school. The gist of his argument was that the purpose of veterinary medicine was not merely to protect that part of the national wealth which the agricultural classes had invested in cattle and horses, but also to protect the human organism from the dangerous accompaniments of cattle epidemics, and for this purpose there was not need of empiricism, but of the *most exact modern science.—Thierärzt. Centralblatt*, February 15, 1896.

At a session of the Lower Austrian Landtag on January 28 a long discussion took place in regard to the swine-plague and the methods of stamping it out. At the conclusion of the discussion the following motion was carried: The Government is petitioned to begin the reorganization of veterinary instruction, so that more attention shall be given to the practical training of veterinarians in treating the diseases of the domestic animals, especially *cattle and swine*.

In reply to this motion the Stadtholder replied that he was happy to announce that a course of instruction had been agreed upon by the Ministers of Education, representatives of the War Department, of the Interior, and of Agriculture, as well as by the veterinary faculties in Vienna and Hamburg and the Austrian Veterinary Association. It was correct that formerly only equine and canine medicine were taught in the veterinary institute; but for a number of years cattle and swine had also been treated, and excursions had been made with the students as far as practicable, in order to make them better acquainted with the diseases of cattle and the needs of the community.—*Thierärztliches Centralblatt*, February 15, 1896.

